# Tissue-Specific Molecular Markers and Heterogeneity in Type 2 Innate Lymphoid Cells

**DOI:** 10.3389/fimmu.2021.757967

**Published:** 2021-10-25

**Authors:** Enrique Olguín-Martínez, Blanca E. Ruiz-Medina, Paula Licona-Limón

**Affiliations:** Departamento de Biología Celular y del Desarrollo, Instituto de Fisiología Celular, Universidad Nacional Autónoma de México, México City, Mexico

**Keywords:** tissue, marker, heterogeneity, ILC2, function

## Abstract

Innate lymphoid cells (ILCs) are the most recently described group of lymphoid subpopulations. These tissue-resident cells display a heterogeneity resembling that observed on different groups of T cells, hence their categorization as cytotoxic NK cells and helper ILCs type 1, 2 and 3. Each one of these groups is highly diverse and expresses different markers in a context-dependent manner. Type 2 innate lymphoid cells (ILC2s) are activated in response to helminth parasites and regulate the immune response. They are involved in the etiology of diseases associated with allergic responses as well as in the maintenance of tissue homeostasis. Markers associated with their identification differ depending on the tissue and model used, making the study and understanding of these cells a cumbersome task. This review compiles evidence for the heterogeneity of ILC2s as well as discussion and analyses of molecular markers associated with their identity, function, tissue-dependent expression, and how these markers contribute to the interaction of ILC2s with specific microenvironments to maintain homeostasis or respond to pathogenic challenges.

## Introduction

Innate lymphoid cells are tissue-resident immune cells derived from lymphoid progenitors. They lack rearranged receptors to recognize specific antigens, therefore, their activation depends on cytokines present in the microenvironment (1). ILCs parallel the heterogeneity observed on T lymphocytes and are classified as their counterparts into cytotoxic NK cells and three main categories of helper ILCs: ILC1, ILC2, and ILC3 (2).

NK cells and ILC1s are subsets of ILCs that can be found in tissues such as liver, skin and gut, among others. They were initially classified within the same ILC1 group because of their expression of IFNγ and NK cell markers like Nkp46 in mice and Nkp44 in humans (1–3). Subsequent analyses determined that unlike helper ILCs, NK cells did not require the GATA-3 transcription factor for their development (4). Furthermore, the cytotoxic activity of NK cells was found to be strongly dependent on the transcription factor Eomes, whereas cytokine production by ILC1s mostly relied on the transcription factor T-bet (5, 6). Together, these findings lead to the separation of the ILC1 population into two independent groups (1, 2, 4–6).

Group 3 innate lymphoid cells were initially only composed of lymphoid tissue-inducer cells (LTis) or Lti-like cells (7–9), until a new cellular subset mainly found in mucosal tissues was identified. This new RORγt-dependent subset played a role in the immune response against infections (10) and together with LTis, it now represents type 3 ILCs (11). ILC3s express cytokines associated with type 3 immunity such as GM-CSF, IL-17 and IL-22 (12, 13).

Type 2 innate lymphoid cells or ILC2s are tissue-resident cells found mainly in the lung, intestine, skin and adipose tissue. Their function depends on the expression of the GATA-3 transcription factor (4, 14) and they are activated by alarmins such as IL-25, thymic stromal lymphopoietin (TSLP) and IL-33 derived from epithelial cells after tissue damage (15–17). ILC2s perform different effector responses in the immune system, both in inflammatory conditions as well as in the maintenance of homeostasis of different tissues.

### Homeostasis of Adipose Tissue

ILC2s are present in white adipose tissue (WAT) where they contribute to homeostasis maintenance through constitutive cytokine secretion. Adipose tissue ILC2s are key producers of IL-5, which is essential for the recruitment and maintenance of eosinophils (18). They also express IL-13, important for the maintenance of M2 macrophages in this tissue (19). Recent work has shown that the expression of inducible costimulator ligand (ICOSL) and OX40 ligand (OX40L) by ILC2s, contributes to the accumulation of a group of regulatory T lymphocytes in visceral adipose tissue and perigonadal adipose tissue respectively (19–21). In addition, the production of methionine-enkephalin peptides from ILC2s induces the expression of uncoupling protein 1 (UCP1) in adipocytes, favoring the browning process of adipose tissue (22), while ILC2-derived IL-13 together with eosinophil-derived IL-4 activate IL-4 receptor α (IL-4Rα) signaling of adipocyte precursors in subcutaneous white adipose tissue to commit them to the beige adipocyte lineage (19, 23).

### Immune Response Against Helminths

ILC2s mediate immunity against helminth parasites through the secretion of type 2 immune response cytokines when they are activated by alarmins such as IL-33 and IL-25 (1, 24, 25). In an infection mouse model using *Nippostrongylus brasiliensis (N. brasiliensis)*, ILC2-derived IL-9 was shown to be indispensable for the expulsion of worms, prompting muscle contraction, goblet cell hyperplasia, and mast cell hyperproliferation (24). In this context, autocrine IL-9, whose expression preceded that of IL-5 and IL-13 (26, 27), increased survival, proliferation, and expression of these cytokines and amphiregulin in lung ILC2s (27). In addition, IL-5-secreting ILC2s are associated with the recruitment of eosinophils, a population that contributes to the elimination of parasites (28). IL-4 and IL-13 derived from ILC2s induce the alternative activation of macrophages and the differentiation of Th2 lymphocytes; in turn IL-13 also acts by inducing smooth muscle contraction and epithelial turnover contributing to worm expulsion (24, 28), in addition to inducing the differentiation of goblet cells and mucus production (24, 28, 29). In the intestinal mucosa, the activation of ILC2s induces hyperplasia of Tuft cells, which increases the production of IL-25, generating a positive feedback loop with ILC2s (25, 29). Lastly, ILC2-derived IL-5 and IL-6 can regulate the production of antibodies by B lymphocytes in fat associated lymphoid clusters during helminth infection of the intestinal and lung barriers (19).

### Tissue Repair

In addition to their roles in inflammation, ILC2s contribute to the maintenance of tissue integrity by inducing repairing mechanisms in damaged tissue after inflammatory processes. They accomplish this through expression of amphiregulin, a member of the epidermal growth factor family (1, 30, 31). Unlike other epithelial growth factor receptor (EGFR) ligands, amphiregulin not only induces a mitogenic signal but is also capable of inducing cell differentiation in a wide variety of cell types in different organs, following a signaling pattern that is like sustained activation of mitogen-activated protein kinases (MAPK) (32). Therefore, ILC2-derived amphiregulin could contribute to tissue repair in a wide range of tissues (32).

### ILC2s in Disease

Allergic diseases such as asthma and atopic dermatitis have also been associated with ILC2s. In murine models of asthma, ILC2s have been observed to be the main source of type 2 cytokines responsible for the hyperproduction of mucus and recruitment of other leukocytes, which together with ILC2s are responsible for asthma symptoms (33, 34). In addition, a group of IL-5 and IL-13-producing resident ILC2s have been observed in the skin of healthy humans and increased in samples from patients with atopic dermatitis, as well as in a murine model of the same condition (15). The presence of hyperactivated ILC2s in other tissues including the nasal mucosa and the intestine has also been described and are linked to the development of pathologies such as allergic rhinitis and food allergy, respectively (35, 36).

Although alarmins have been reported to be the main activators of ILC2s, these cells can also respond to cytokines derived from the immune compartment like IL-2 and IL-7 (37). Recent studies have also shown a plethora of different molecules, besides cytokines, that can positively or negatively regulate the functions of ILC2s in different contexts, both *in vitro* and *in vivo*. Among these stimuli are lipid mediators (leukotrienes and prostaglandins), neuropeptides, hormones and diet components (38), demonstrating that the regulation of ILC2 function is tightly controlled by a variety of signals within the cellular microenvironment.

Recent studies in mice and humans suggest that the ILC2 population is extremely diverse. Markers typically used for their identification exhibit different expression profiles in a tissue-dependent fashion. Additional tissue-specific heterogeneity is found in ILC2 activation (39–44).

This review focuses on the molecular markers that provide ILC2s their identity and the receptors that regulate their function *in vivo*. We will discuss the phenotypic features and signaling pathways activated in specific tissues and how these allow ILC2s to interact with and be regulated by the environment within which they are embedded.

## Receptors of Epithelial Cell Derived Cytokines

**Figure 1:**
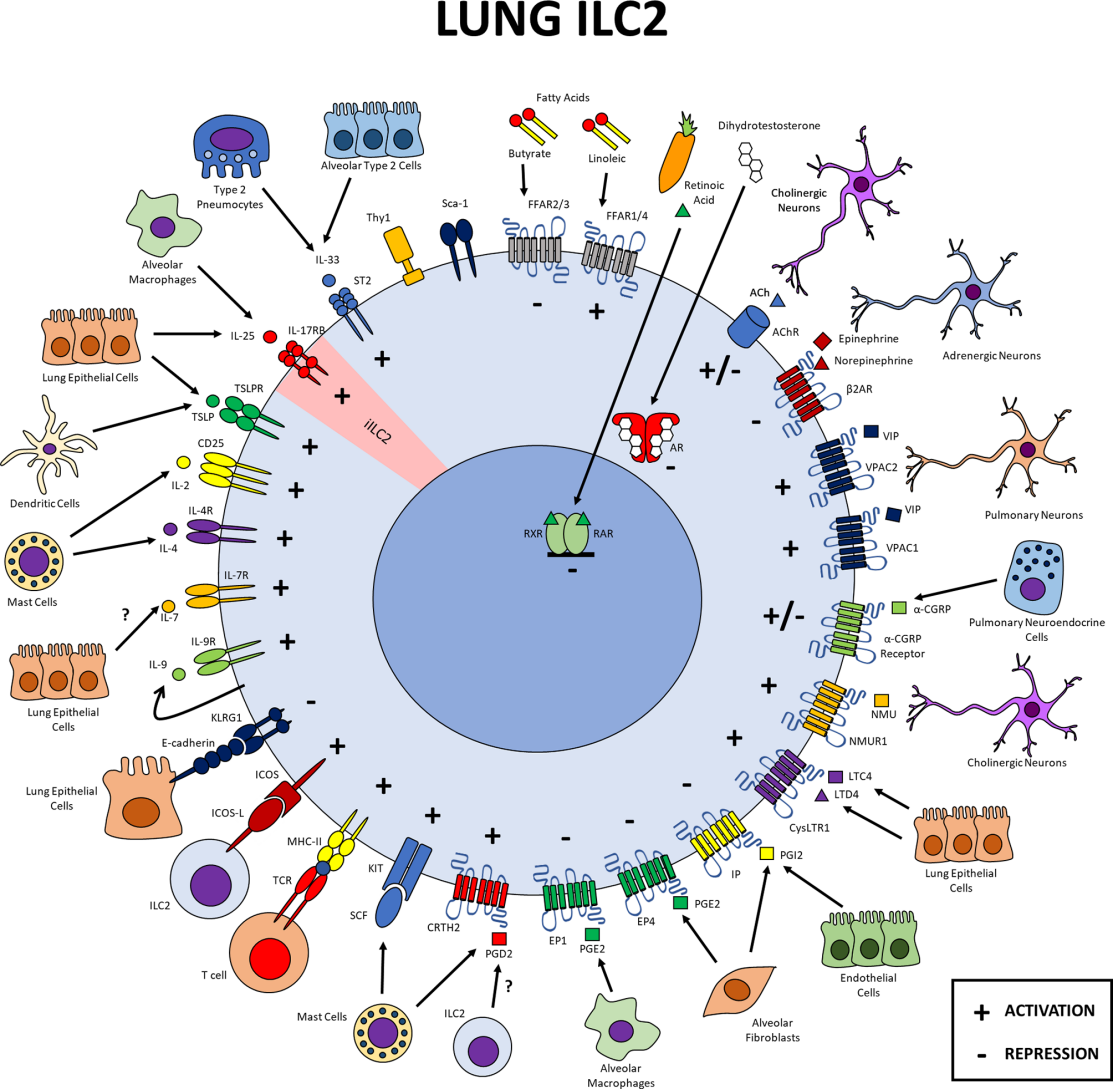
Lung ILC2s. Expression of different markers on pulmonary ILC2s and possible cellular interactions within this tissue. Lung ILC2s interact with the epithelium and different cell populations of the immune and the nervous systems. Cytokine receptors transduce signals that activate ILC2s, as does the immune checkpoint molecule ICOS and MHCII. Lipid mediators PGD2, LTC4 and LTD4 induce positive signals in ILC2s while PGE2 and PGI2 negatively regulate their function. The neuropeptides NMU and VIP positively regulate ILC2s while catecholamines drive a negative regulation and CGRP and ACh displays a dual function. Dihydrotestosterone and Klrg1 repress lung ILC2s. Diet derived factors like retinoic acid and butyrate inhibit ILC2 function while linoleic promotes it. iILC2 inflammatory ILC2, SCF stem cell factor, PG, prostaglandin; LT, leukotriene; NMU, neuromedin U; CGRP, calcitonin gene related peptide; VIP, vasoactive intestinal peptide; ACh, acetylcholine; RAR, retinoid acid receptor; RXR, retinoid X receptor.

**Figure 2:**
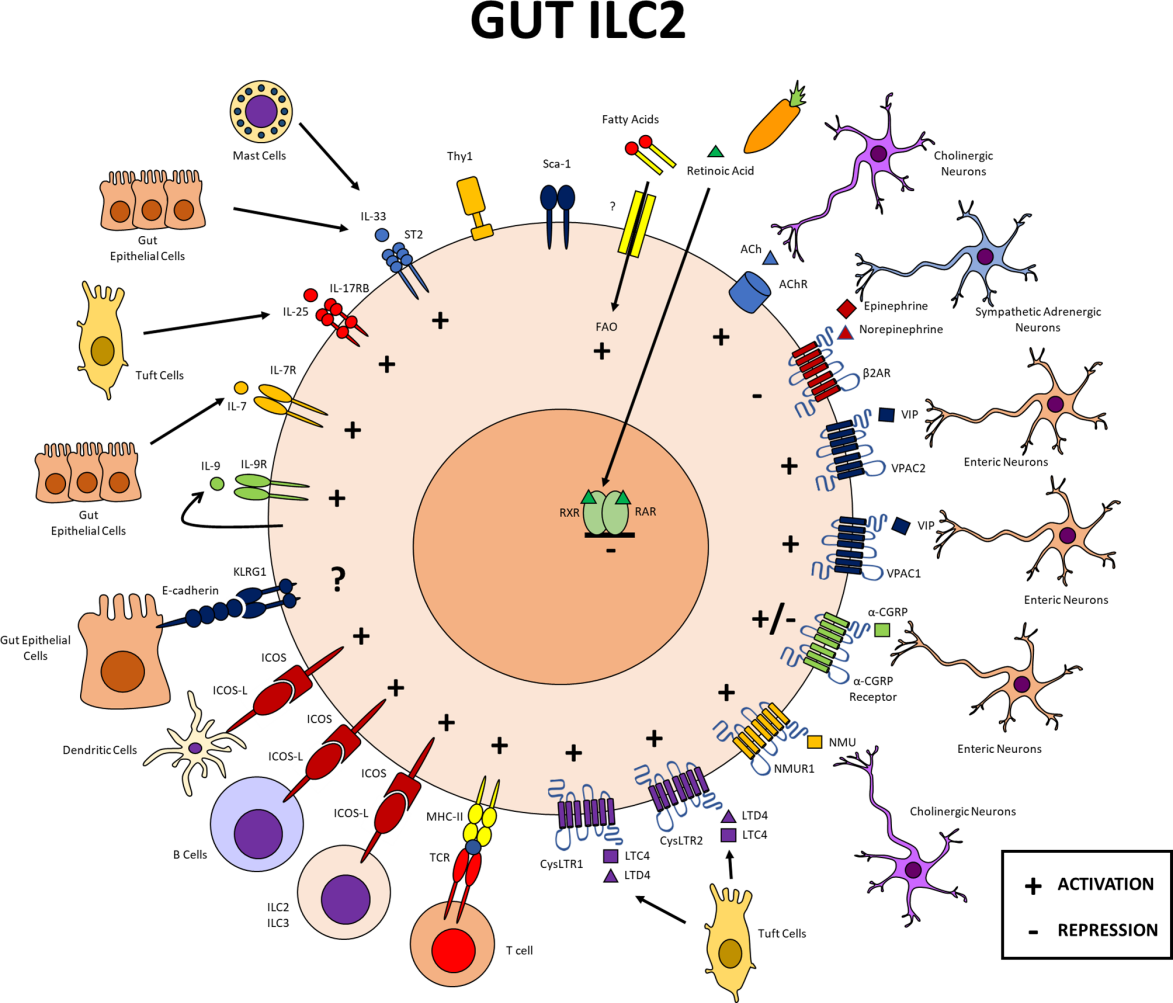
Gut ILC2s. Expression of different markers in intestinal ILC2s and cellular interactions within this tissue. In the gut, ILC2s interact with other immune cells, neurons, epithelial cells, among other populations. All cytokine, leukotriene, and neuropeptide receptors mentioned in this review, except the β2AR, provide signals that activate ILC2s in the gut. CGRP both positively and negatively regulates these cells. In the gut, expression of MHCII, ICOS and its ligand favors the function of ILC2s. The function of ILC2s in this tissue depends on fatty acid oxidation, while retinoic acid inhibits ILC2s. PG, prostaglandin; LT, leukotriene; NMU, neuromedin U; CGRP, calcitonin gene related peptide; VIP, vasoactive intestinal peptide; RAR, retinoid acid receptor; RXR, retinoid X receptor; fatty acids oxidation.

**Figure 3:**
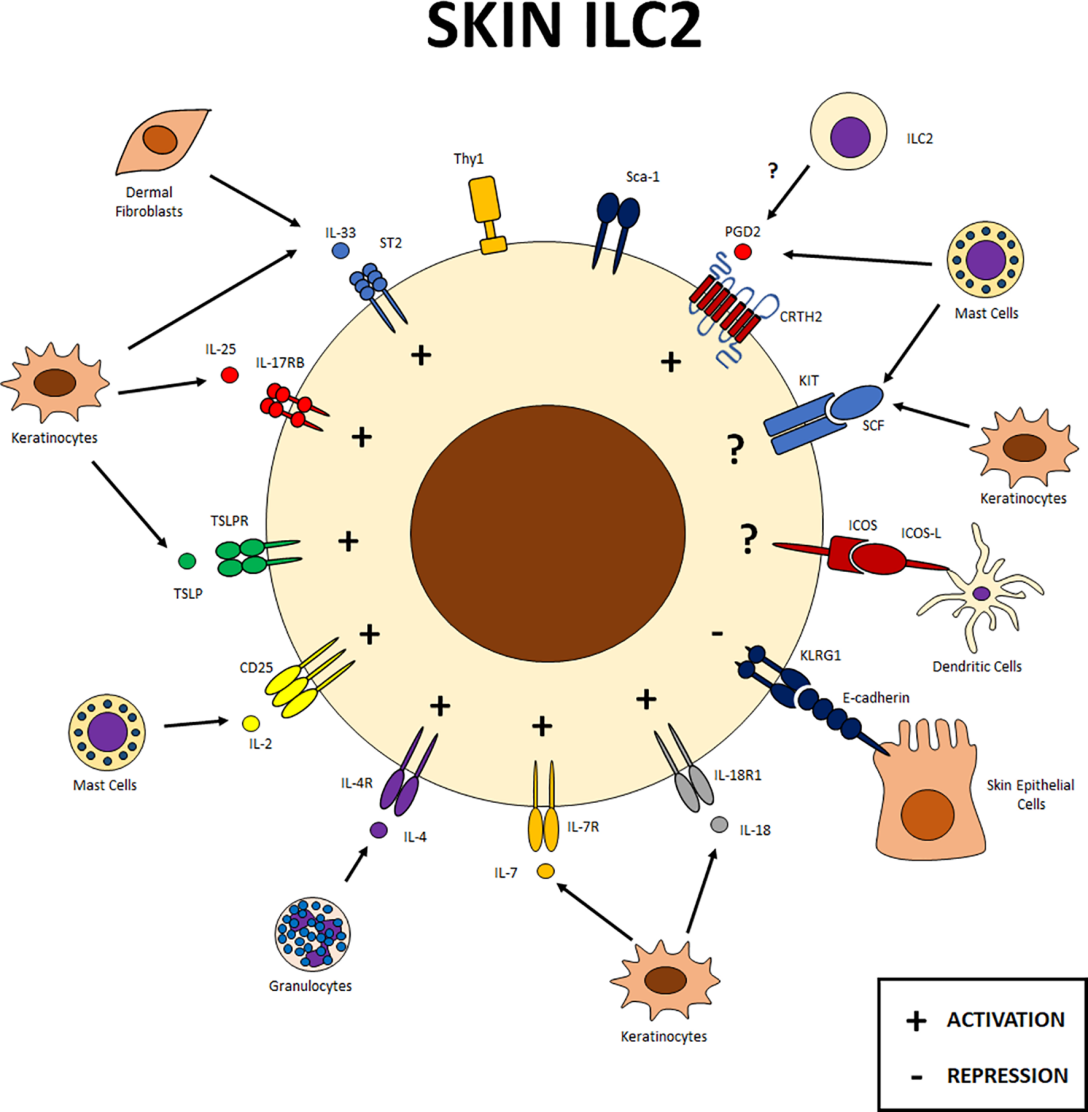
Skin ILC2s. Expression of different receptors on skin ILC2s and cellular interactions within this tissue. ILC2s in the skin interact with different cell populations, including epithelial cells and other components of the immune system. Signals from IL-33, IL-25, TSLP, IL-2, IL-4, IL-7, IL-18 and PGD2 receptors favor ILC2 function in this tissue. Klrg1 on ILC2s can interact with E-cadherin to negatively regulate these cells in the skin. Other receptors such as KIT and ICOS have also been reported in skin ILC2s although their specific function has yet to be determined. SCF, stem cell factor; PG, prostaglandin; LT, leukotriene.

Barrier epithelial cells produce a variety of cytokines that can modulate the immune system. These include IL-33, IL-25 and TSLP, a prototypical group of cytokines produced in response to specific stimuli such as tissue damage by allergen exposure, and helminth infection, which induce type 2 immune responses. These cytokines activate ILC2s (**Figures 1**–**3**), among different cells of the immune system, to induce their expansion, survival and expression of cytokines (45–47).

### Suppressor of Tumorigenicity 2

ST2 was recognized as one of the first markers for the identification of ILC2s. ST2 also known as IL-1 receptor like 1 (IL1RL1) dimerizes with IL-1 receptor accessory protein (IL-1RAcP) to form the IL-33 receptor. Once activated by IL-33, the receptor signals through myeloid differentiation primary response gene 88 (Myd88), interleukin-1 receptor-associated kinases 1 and 4 (IRAK1 and IRAK4) and tumor necrosis factor receptor associated factor 6 (TRAF6) to trigger the activation of MAPK and Nuclear Factor kappa B (NFkB) (48). When activated, ST2 induces the proliferation of ILC2s and their secretion of IL-4, IL-5, IL-9 and IL-13 in a tissue, species and microenvironment-dependent manner (38).

In mice, ST2 is expressed in several immune cells including T cells (49, 50), macrophages (51), basophils and mast cells (52) among others. On ILC2s, it is mainly expressed in the lung, although it can also be found in bone marrow and adipose tissue, while its basal expression is limited in tissues such as the gut and skin (39, 41).

In the lung, IL-33 participates in the activation of most of the inflammatory processes in which ILC2s are involved (**Figure 1**); it does so by inducing the expression of IL-5 and IL-13, mainly in models of upper airway inflammation or helminth infection (53–55). In a sepsis model, ILC2 activation induced by IL-33 was reported to be important for maintaining epithelial cell viability through the secretion of IL-9, which inhibits pyroptosis of epithelial cells (56).

In WAT, the expression of ST2 on ILC2s is of great importance. IL-33/ST2 signaling activates ILC2s, maintaining tissue homeostasis and preventing obesity in mice and humans. This signaling is also important in the biogenesis of brown adipose tissue and therefore in thermoregulation. These processes seem to be regulated by the secretion of the cytokines IL-5 and IL-13, as well as methionine-enkephalin peptides, which directly induce the browning of adipocytes in WAT (18, 22, 23, 57).

ST2 expression in ILC2s and its precursors has also been reported in the bone marrow. IL-33 induces ILC2 egress from the bone marrow to populate tissues in the perinatal period of mice (58). In addition, ST2 signaling in response to IL-33 allows for local expression of ILC2-derived IL-5 in the bone marrow, which induces eosinophilia and promotes inflammation (59).

ILC2s from the small intestine and colon express ST2 in mice at steady state (**Figure 2**) (60–62). This expression is also seen in mesenteric lymphoid nodes where IL-33 induces a recently described population of inflammatory ILC2s (iILC2s) characterized by decreased expression of ST2 and CD25 (62). In the small intestine, IL-33 also induces iILC2s and upregulation of tryptophan hydroxylase 1 which results in decreased susceptibility to infection by helminths (62). In the colon, ILC2s expressed ST2 after intraperitoneal administration of IL-33, leading to their proliferation, a high expression of IL-5 and IL-13, and low IL-17 levels (63).

There are some reports on the effects of IL-33 in the skin and its participation in wound healing. However, the specific mechanism responsible for this function has not been elucidated (**Figure 3**), although it could be explained by the known tissue repairing properties of ILC2s (64). IL-33 has been reported in skin-associated pathologies such as atopic dermatitis and psoriasis (16, 65, 66). The relevance of IL-33 in the immune response against helminth infection in humans remains unclear, possibly due to the limitations that exist in studying human ILC2s. Nevertheless, some studies suggest that this pathway works similarly to skin pathologies and adipose tissue homeostasis in mice (16, 22).

ILC2s also reside in the central nervous system (brain, spinal cord and meningeal space) under steady state; these ILC2s express ST2 and expand in response to IL-33 (67, 68). Brain meningeal ILC2s increase their expression of IL-5 and IL-13 in response to spinal cord injury in an IL-33-dependent manner and contribute to recovery (67). Additionally, in an experimental autoimmune encephalitis model, IL-33 activated CNS ILC2s ameliorate the disease by antagonizing the Th17 response (68).

IL-33 is an alarmin released in conditions of tissue damage. Hence, expression of ST2 on ILC2s within tissues such as skin and lung that are damaged during a parasitic infection or allergic process is crucial and provides a key mechanism for restoring homeostatic conditions. Additionally, the relevance of the ST2/IL33 axis in the control of other physiological responses is demonstrated by the specific functions it regulates in adipose tissue, where IL-33 derived from stromal cells coordinates key developmental and adaptational processes (69, 70).

### IL-17 Receptor B

IL-17RB together with IL-17RA conform the receptor for IL-25 (IL-17E) (71), an alarmin of the IL-17 family that activates ILC2s. Studies evaluating the heterogeneity of the ILCs have found IL-17RB to be a marker almost exclusively restricted to the gut; however, some expression has also been observed in the lung (39, 41).

ILC2s express higher levels of IL-17RB in the small intestine (**Figure 2**) compared to other tissues such as lung, skin and fat; this allows a communication with Tuft cells that produce IL-25 and enables ILC2 expression of IL-5 and IL-13 in homeostasis. Furthermore, Tuft cell numbers increase during helminth infections, in turn secreting more IL-25 to promote a more efficient activation of ILC2s during the defense response against these pathogens (72–75).

In the lung, expression of IL-17RB has been described in a particular population: inflammatory ILC2s (iILC2s) (**Figure 1**). These cells are characterized by low ST2 and high KLRG1 expression and can produce IL-5 and IL-13 in response to IL-25. In different *in vitro* and *in vivo* models, iILC2s give rise to natural ILC2s (nILC2s), which are characterized by higher expression of ST2 (17). However, it has also been reported that IL-33 stimulation can promote nILC2s to become iILC2s, thus generating a population that is important for the correct immune response against helminth infection (62).

IL-17RB-expressing ILC2s are also relevant in the skin (**Figure 3**). An increased expression of this receptor on ILC2s has been reported in patients with atopic dermatitis (16). Additionally, in mice, keratinocyte-derived IL-25 activates ILC2s, promoting IL-13 expression, Th2 cell accumulation and epidermal hyperplasia in allergic skin inflammation (76).

### TSLP Receptor

TSLP is a pleiotropic cytokine originally isolated from a murine thymic stromal cell line and characterized as a lymphocyte growth factor (77). It was later shown to activate ILC2s and the expression of the TSLP receptor has been used as a marker to identify these cells. TSLP binds to its receptor TSLPR and forms a complex that subsequently binds to the IL-7 receptor alpha chain (IL-7Rα) to initiate a JAK/STAT5 dependent signaling pathway (77).

Although the transcript for this receptor has been detected in murine ILC2s from lung, gut, adipose tissue, skin and bone marrow (39), there are no reports directly showing the expression of the TSLP receptor at the protein level in any of these tissues except for the lung in mice (78, 79) and the skin in humans (16).

In the lung, ILC2s respond to TSLP (**Figure 1**) in allergy and viral infection. TSLP is important to potentiate the effects of IL-33 on ILC2s such as proliferation and IL-5 and IL-13 production (46, 78, 79). In addition, TSLP can promote ST2 expression on ILC2s; in turn, IL-33 increases TSLPR expression, initiating a positive feedback loop potentiating the alarmin responsive capacity of ILC2s (79). Regardless of IL-33, TSLP plays an essential role in papain-induced airway inflammation by stimulating ILC2s *in vivo* to promote production of type 2 cytokines (80).

In the skin, ILC2s have been reported to be important players in the induction of inflammatory processes such as atopic dermatitis in both humans and mice. In a murine model of dermatitis, an IL-33-independent inflammatory process has been reported, where TSLP is essential for the induction of ILC2 proliferation (15). In humans TSLP induces CD1a expression in the skin, which promotes the production of IFNγ, IL-22 and IL-13 by CD1a-reactive T cells, and a defense response against some types of bacteria (81).

TSLP is primarily expressed in lung and gut epithelial cells, as well as in skin keratinocytes (77); consequently, the role of this cytokine in ILC2s from these tissues is being actively investigated. Most reports agree that TSLP works in cooperation with IL-33 to enhance cytokine expression and proliferation responses in ILC2s in the lung. In the skin, TSLP appears to play an important role since it induces responses independent from other alarmins (46, 78, 79). Interestingly, there are not yet to be reports on the function of TSLP in the gut.

## γ-Common Cytokine Receptors

IL-2, IL-4, IL-7, IL-9, IL-15 and IL-21 are members of a family of cytokines that regulate biological functions in different cell types of the immune system. They share the common gamma chain (γc) receptor, a subunit initially discovered as the third component of the IL-2 trimeric receptor (IL-2Rγ or CD132) (82). ILC2s have been reported to be a major source of IL-9 and under certain stimuli can also produce IL-4. They have also been shown to express IL-9 and IL-4 receptors, suggesting a potential autocrine regulation of these mediators on the cells. Specific subunits of the IL-2 and the IL-7 receptors belonging to this family of cytokines are used as markers for ILC2s on different tissues.

### IL-2 Receptor Alpha

The high affinity IL-2 receptor is made up of 3 subunits: IL-2Rα (CD25), IL-2Rβ (CD122) and the γc chain (CD132). IL-2 binds to CD25 and this complex recruits CD122 and CD132 to initiate signaling leading to STAT5 activation (83).

CD25 is used as a marker for ILC2s identification in most tissues in mice, including bone marrow, lung, gut, adipose tissue and skin (39–41). In humans, it is found on ILC2s from the lung, tonsils, spleen, bone marrow and peripheral blood (42, 43).

The expression levels of CD25 in the lung vary depending on the *in vivo* model used. IL-33-activated ILC2s uniformly express high levels of CD25, while exposure to house dust mite (HDM) generates a model of allergic inflammation in which low expression of CD25 is observed on ILC2s without affecting their ability to produce type 2 cytokines (84). CD25-expressing ILC2s have also been reported to proliferate in response to IL-2 produced by mast cells, thus contributing to lung inflammation in allergy models (85). In humans, it has been observed that retinoic acid increases the expression of CD25 among other markers, and in combination with IL-2 induces these cells to acquire a regulatory phenotype by promoting the expression of inhibitory receptors such as CTLA4 and cytokines such as IL-10 (86).

In the skin, CD25 is expressed by ILC2s (**Figure 3**) and this expression increases in models of allergic inflammation (87). Dermal ILC2s require IL -7 for survival and can respond to IL-2, resulting in production of IL-5, and contributing to the induction of dermatitis (88).

Finally, recent reports suggest that intrahepatic ILC2s express CD25 both basally and in inflammatory conditions, allowing this subset to be activated by T lymphocytes, the intrahepatic sources of IL-2 (89, 90).

### IL-4 Receptor Alpha

IL-4 binds its receptor IL-4Rα to form a complex that subsequently recruits the γc chain in lymphoid cells, or the IL-13Rα1 in myeloid or non-hematopoietic cells. In both cases, activation of the IL-4 receptor complex leads to signaling dependent on STAT6 activation (91).

The IL-4 receptor alpha and the γc chain are expressed in mouse and human lung ILC2s (**Figure 1**) (92–94). *In vitro* stimulation of murine ILC2s with IL-4 increases the expression of IL-5, IL-13, IL-9, CCL11, CCL5 and CCL3 induced by IL-33 and IL-2. In a papain-induced inflammation lung model, deletion of IL-4 specifically in basophils results in decreased numbers of ILC2s and their production of IL-5 and IL-13, along with diminished eosinophilic inflammation induced by protease allergens (93).

Skin ILC2s also express IL-4Rα (**Figure 3**) and the proliferation and inflammatory responses of these cells are dependent on basophil-derived IL-4 in a murine model of atopic dermatitis (95).

The IL-4 receptor is also expressed in peripheral blood ILC2s in humans. Stimulation with this cytokine is important for the increase in ILC2 numbers and the maintenance of the CRTH2 expression marker (94). CRTH2 acts as the PGD2 receptor whose activation cooperates with IL-25 and IL-33, enhancing ILC2 functions such as migration and expression of Th2 cytokines (96, 97).

As described in these reports, the main sources of IL-4 are mast cells, basophils, eosinophils, T lymphocytes and under specific stimuli, ILC2s (91). Importantly, these cell populations partner with ILC2s in type 2 immune inflammatory responses.

### IL-7 Receptor Alpha

IL-7Rα or CD127 forms the IL-7 receptor in conjunction with the γc chain. IL-7Rα is also a subunit of the TSLP receptor. Given that the importance of TSLP was previously discussed, we will mainly focus on the relevance of IL-7Rα as an IL-7 receptor on ILC2s in this section.

IL-7 is necessary for the expression of NFIL3, a transcription factor required for the expression of Id2 and the generation of the common helper innate lymphoid progenitors (CHILP) (98). IL-7 contributes to the development of more committed precursors to certain ILC lineages, however the molecular mechanisms involved are currently unknown. CD127 is expressed in all helper ILC populations, and a recent study suggests that this expression works as a mechanism to regulate IL-7 activity in other lineages by restricting its availability in the niche (99). CD127 expression in the ILC2 group has been reported in several tissues including lung, gut, skin (**Figures 1**–**3**), and adipose tissue, among others (39–44, 100).

Besides the role of IL-7 in the development of ILC2s, this cytokine can control other cellular functions in specific tissues. For example, in the stomach, an organ in which ILC2s have been recently described, CD127 expression on these cells is higher compared to other tissues. In addition, ILC2s can respond to IL-7 both *in vitro* and *in vivo* by proliferating and producing IL-5 and IL-13 (101).

In the lung, CD127 is critical for the induction of natural and inflammatory ILC2s. Mice deficient in this receptor showed a complete absence of ILC2s, exhibiting the same phenotype observed in mice deficient of the γc chain (17). These data suggest that the phenotype observed in those mice could be associated with a deficiency in IL-7 signaling. Accordingly, IL-7 or γc chain deficiency in the skin also results in a total absence of dermal ILC2s (88).

IL-7 is expressed in both immune and non-immune cells including stromal cells, keratinocytes, gut epithelial cells, follicular dendritic cells, macrophages, monocytes and B cells (102); therefore, they could serve as primary sources of IL -7 for ILC2s in different tissues.

### IL-9 Receptor Alpha

IL-9 is a pleiotropic cytokine associated with classical type 2 immune responses. Overexpression of this cytokine is associated with mastocytosis, eosinophilia, increased production of mucus, airway hyperreactivity and resistance to helminth infections (103, 104). Binding of IL-9 to IL-9Rα induces the heterodimerization of this receptor with the γc chain, leading to activation of Jak1 and Jak3 kinases, which induces receptor phosphorylation and activation of STAT1, STAT3 and STAT5 (103).

Lung ILC2s express high levels of the IL-9 receptor (**Figure 1**) in a context of airway inflammation in a papain administration model where stimulation with this cytokine potentiates the expression of IL-5, IL-6 and IL-13 in these cells (105). In an infection model with *N. brasiliensis*, ILC2s highly express IL-9 (27, 106), while mice deficient in IL-9 receptor have reduced numbers of ILC2s as well as impaired cytokine expression, deficient parasite clearance and inefficient repair of damaged tissue (27). Lastly, stimulation of lung ILC2s with IL-9 *in vitro* increases their survival and expression of IL-5 and IL-13 (27), indicating the importance of this cytokine on ILC2 function.

Expression of IL-9Rα on ILC2s has been reported in the small intestine (**Figure 2**), however, its direct function has not been studied (41, 107).

ILC2s are characterized by expressing IL-9. In fact, the main source of IL-9 in models of lung inflammation with papain are ILC2s themselves (105). In addition, ILC2s are the main source of IL-9 in the resolution phase of arthritis and the deficiency of this cytokine reduces the numbers of ILC2s (108). Finally, in helminth infection, both ILC2 and Th9 cells can produce this cytokine in the lung and small intestine (104). These reports suggest an autocrine regulation of the ILC2 functions mediated by IL-9.

## Other Cytokine Receptors: IL-18 Receptor 1

A recent study aimed to determine the tissue-specific expression profiles of ILC2s in mice through single cell RNAseq analysis, revealed the presence of the IL-18 receptor 1 (a cytokine of the IL-1 family) specifically in skin ILC2s (39).

IL-18 is important for the *in vivo* function of ILC2s. Mice deficient for this cytokine challenged with MC903 exhibit defects in the proliferation and activation of skin ILC2s (39). Furthermore, ILC2 skin cultures showed increased expression of IL-5 and IL-13 in the presence of IL-18 (39). Although IL-18R1 expression was also observed in a small population of lung ILC2s, the difference in the frequency and expression of this marker suggests a greater relevance of IL-18 signaling in skin ILC2s (**Figure 3**).

## Cell-Cell Interaction Molecules

Since their discovery, ILC2s have been characterized for expressing different surface molecules that allow them to interact directly within their group and with other cells in the microenvironment (109). These interactions play an essential role in the regulation of ILC2 function in different inflammatory contexts.

### Killer Cell Lectin-Like Receptor Subfamily G Member 1

Klrg1 is important for ILC2 cell-cell interaction and is used as a marker to identify these cells *in vivo*. It is an inhibitory receptor originally associated with NK cells and able to interact with proteins of the cadherin family (110). Several studies have described the expression of Klrg1 on ILC2s from different tissues in both mice and humans. In mice, Klrg1 transcripts have been reported in lung, gut and adipose tissue (39–41); while in humans, this molecule has been reported in blood, bone marrow, spleen, lung, tonsils and colon (42–44).

In murine models, Klrg1 has been described as a maturation marker in small intestine ILC2s (**Figure 2**), identifying IL-5 and IL-13 producers in this tissue (107). In the lung, Klrg1 expression has been reported in basal conditions (**Figure 1**) (111, 112). Its role as a negative regulator was recently demonstrated in irradiated mice that received bone marrow from Klrg1-deficient mice together with wild type bone marrow. Klrg1-deficient ILC2s presented a competitive advantage, increasing their ratio in the lung with no differences observed in their proliferation or cytokine production compared to wild type ILC2s (113). Upon IL-25 stimulation, a distinct population of inflammatory ILC2s whose expression of Klrg1 is much higher, emerges. The induction of this population has been observed at specific time points in the lung during models of helminth infection, where they orchestrate the immune response against this class of pathogens (17, 114).

Regarding the skin, Klrg1 expression has been observed under allergic conditions (**Figure 3**) (87). Patients with atopic dermatitis show increased expression of Klrg1 and the interaction of activated ILC2s with E-cadherin results in a down regulation of GATA-3, IL-5, IL-13, amphiregulin and reduced ILC2 proliferation; suggesting a negative regulatory role for Klrg1 in skin ILC2s (16). In bone marrow derived ILC2s, Klrg1 deficiency restores the decreased proliferation observed by the effect of E-cadherin (113). This suggest that Klrg1 could have the same function in other tissues, especially considering that E-cadherin is a protein widely expressed in all epithelial tissues.

### The Inducible T Cell Costimulator

ICOS is a costimulatory molecule belonging to the CD28 superfamily (115) whose expression has been associated with different populations of immune cells. This molecule is used as a marker for ILC2s in different models.

ICOS expression in mouse ILC2s has been reported in tissues such as bone marrow, lung, adipose tissue, gut and skin (39). In humans, the expression of ICOS seems to be restricted to tissues such as the lung, tonsils and skin (42).

In the lung and small intestine, signaling mediated by ICOS and its ligands is important for ILC2 survival and proliferation at steady state and inflammation; in this last condition, it is also important to produce IL-5 and IL-13 (116–118). In the lung, ILC2s express the ICOS ligand (ICOS-L), thus providing a path to interact with each other and enhance their activation (**Figure 1**) (117). Although its role has not been studied in skin, ICOS has been shown to be expressed on ILC2s on this tissue (**Figure 3**) (119) and increased in allergic conditions (87).

Similarly, a specific function for ICOS has not been described in adipose tissue, however, visceral adipose tissue ILC2s express ICOS-L and interact with regulatory T cells to help maintain homeostasis (20).

ICOS and ICOS-L expression is also detected in human peripheral blood ILC2s, along with an increased presence when these cells are activated (117). Additionally, there are reports of ICOS expression on ILC2s from nasal polyps which is reduced in conditions of chronic rhinosinusitis; however, its specific function there has not been determined (120).

Altogether, the expression of ICOS and ICOS-L in different tissues suggest that the function of this marker might be to enhance ILC2 activation and allow communication within that group as well as with other cells in different tissues, including regulatory T cells and dendritic cells.

### Class II Major Histocompatibility Complex

Antigen presentation by MHC-II molecules is critical for the maintenance of self-tolerance and the initiation of an effective immune response. These molecules are constitutively expressed on professional antigen presenting cells (APCs) such as dendritic cells, macrophages, B cells and thymic epithelia (121). Recent studies have shown that ILC2s are capable of presenting antigens and the expression of MHC-II in these cells has been reported in tissues such as the lung, small intestine and colon (63, 74, 105, 122, 123).

A fraction of lung ILC2s express high levels of MHC-II (**Figure 1**) and present antigens, inducing T cell proliferation and differentiation towards a Th2 phenotype. In turn, T cells secrete IL-2, inducing ILC2 proliferation and expression of IL-5 and IL-13 (122). Expression of MHC-II in lung ILC2s is also induced during *N. brasiliensis* infection and is dependent on STAT6 activation (123).

In the gut, MHC-II expression has been reported in the small intestine and colon (**Figure 2**) (63, 74). Small intestine ILC2s express MHC-II in *Trichinella spiralis* infection, which allows their interaction with CD4+ T cells for the induced expression of type 2 cytokines in response to IL-25 (74).

In humans, increased expression of MHC-II has been reported in peripheral blood ILC2s of patients with acute exacerbation of chronic obstructive pulmonary disease (AECOPD). Cocultures of these ILC2s with Th2 cells showed an increased expression of IL-4, IL-5 and IL-13 that is dependent on MHC-II-mediated interaction (124).

Hence, the MHC-II-mediated interaction between T lymphocytes and ILC2s plays an additional role in the immune response against helminth parasites. IL-2 derived from T cells induces proliferation of ILC2s and enhances the expression of type 2 cytokines, contributing to the elimination of parasites and demonstrating a cooperation between these two cell populations (125).

### Growth Factor Receptor: KIT

The stem cell factor (SCF) also called mast cell growth factor or KIT-ligand was described several years ago as being of great importance in the physiology and pathology of the skin. Its receptor, KIT, is expressed in mast cells and melanocytes (126). Subsequent studies reported this ligand-receptor pair in other tissues and currently the expression of KIT has been widely described in different groups of ILCs.

KIT is a type 3 tyrosine kinase receptor. Interaction with its ligand occurs in different tissues where it can regulate processes such as cell survival, proliferation, migration and differentiation (127). This receptor is expressed on ILC2s and ILC3s. On ILC2s, it is expressed in different tissues like lung and skin; however, its function has not been fully characterized in all of them (42).

KIT is expressed in human peripheral blood ILC2s, where it allows the distinction between two cell populations. ILC2s with higher levels of KIT expression acquire the ability to secrete cytokines associated with ILC3s, while the ones with low expression express high levels of type 2 cytokines, possibly corresponding to mature and lineage committed ILC2s (128, 129). These KIT+ cells are increased under conditions of helminth parasite infections and can also produce IL-13 (130). TSLP, IL-25 and IL-33 signaling *in vitro* can promote KIT expression on ILC2s (131).

In mice, KIT is also heterogeneously expressed on ILC2 populations in the lung, exhibiting high or low expression of this marker in the context of allergies and viral infections where these cells are important producers of IL-5 and IL-13 (33, 132–134). SCF is also important for the increase of ILC2 numbers in a context of allergic inflammation, and induces the expression of IL-4, IL-5, IL-9, IL-13 and TGFβ in these cells (132).

In humans, heterogeneous KIT expression is observed under homeostatic conditions in the skin (**Figure 3**), and cells expressing this protein increase in patients with psoriasis, concomitant with the acquisition of an ILC3 phenotype (129); however, dermal ILC2s maintain their potential since upon stimulation with IL-33 and TSLP they are still capable of producing IL-13 (135). In addition, ILC2s from peripheral blood that express KIT also express RORγt, although at lower levels than ILC3s, and can produce IL-17. Furthermore, a fraction of this cells has been found to express skin homing markers such as CCR10 and cutaneous lymphoid antigen (CLA) (129, 135); therefore, it is proposed that KIT^+^ ILC2s could migrate to the skin and contribute to chronic inflammation in pathological conditions such as psoriasis (129).

Expression of KIT has also been described in other mice tissues. The first reports of ILC2s in fat-associated lymphoid clusters were phenotypically defined as KIT positive cells, able of express type 2 cytokines important in the defense against helminth parasites (37). KIT expression in functional ILC2s has also been shown in mesenteric lymph nodes, spleen, liver, and corneal limbus (136–138).

Expression of the KIT ligand is associated with different cell types including fibroblasts, keratinocytes (126) and stromal cells. In humans, this factor is found in mast cells from lung and skin (139), suggesting that ILC2s could interact with these cells through this signaling pathway.

## Lipid Mediator Receptors

In addition to the classic activators of ILC2s, it has been shown that lipid mediators like prostaglandins and leukotrienes derived from arachidonic acid can regulate the function of these cells by positively or negatively controlling their activation (140, 141).

### Chemoattractant Receptor-Homologous Molecule Expressed on Th2 Cells

CRTH2, the receptor for prostaglandin D2, is one of the most common prostaglandin receptors used for ILC2 identification in humans. CRTH2 is a Gi protein coupled receptor that regulates the function of cells by lowering cAMP levels and inducing calcium mobilization (142).

In humans, CRTH2 positive ILC2s can be found in fetal and adult lung and intestinal tissues, as well as in adult nasal polyps and peripheral blood (143). Another report indicates that this marker is highly expressed in human and murine peripheral blood ILC2s in the context of helminth infections or upon activation with alarmins, where the CRTH2/PGD2 pathway is important for the accumulation of ILC2s in the lung (**Figure 1**) (144, 145).

Teunissen et al. described the presence of CRTH2 positive ILC2s in healthy human skin samples (**Figure 3**). It has also been reported that CRTH2+ ILC2s from peripheral blood express skin-homing markers such as cutaneous lymphocyte antigen (CLA) (135). PGD2 is important in skin where it acts as a synergic signal with IL-25 and IL-33 for the migration and induction of cytokine expression (96). Ex vivo activation of skin ILC2s with PGD2 induces the expression of cytokines associated with the type 2 response as well as pro-inflammatory cytokines such as IL-3, IL-8, IL-21, GM-CSF and CSF-1. IL-3, GM-CSF and CSF-1 contribute to myeloid cell differentiation, while IL-8 and IL-21 have been associated with the recruitment of neutrophils. Together, these signals could contribute to the allergic inflammation induced by ILC2 (96).

The CRTH2 pathway has also been reported in lymphoid tissue. Interestingly, tonsillar ILC2s express not only the CRTH2, but can also produce PGD2 when activated by IL-2, TSLP, IL-25 and IL-33. This apparent autocrine loop has been suggested to be essential for the expression of IL-5 and IL-13 and for the increased expression of GATA-3 and CD25 on these cells (97).

CRTH2 expression in murine ILC2s has been described in the lung (**Figure 1**); however, this receptor is more commonly used in the analysis of human ILC2s and consequently, most of the evidence showing the importance of the expression of this marker is restricted to human studies. Regardless of whether CRTH2 is important for murine ILC2 regulation or not, a common feature in both species is that one of the main sources of PGD2 are mast cells, providing a potential direct interaction between those two cell types. Other sources of PGD2 such as Tuft cells have been characterized in the small intestine in helminth infection (146) or dendritic cells in skin (147). Finally, the interesting observation that ILC2s can produce PGD2 themselves also hints to a possible autocrine function of this signal on ILC2s that requires further characterization. These results show that through the PGD2-CRTH2 pathway, ILC2s can interact with different immune cells at different anatomical sites to be activated, thus enhancing their effector functions in the type 2 immune response.

### E-Type Prostanoid Receptors (EP1, EP2 and EP4)

Three out of the four existing prostaglandin E2 (PGE2) receptors have been reported to be expressed on ILC2s (148, 149). These receptors are coupled to different G proteins. EP1 is associated with Gq proteins and therefore induces calcium mobilization while EP2 and EP4 are associated with Gs proteins, hence promoting increases in cAMP (150). Tonsillar and peripheral blood human ILC2s express EP2 and EP4 receptors. EP2 and EP4 play a protective role in the lung during allergic inflammation due to their inhibitory effect on ILC2 proliferation and GATA-3 and CD25 expression, as well as in IL-5 and IL-13 induction, all of this in response to activation with IL-2, TSLP, IL-25 and IL- 33 (149).

EP1 and EP4 transcripts have been observed in ILC2s isolated from murine lung (**Figure 1**). In addition, an EP4-dependent inhibition induced by prostaglandin E2 has been reported in ILC2s upon activation induced by IL-33 *in vitro* (148). Furthermore, it was observed that the administration of PGE2 attenuates inflammation induced by IL-33 *in vivo*, affecting the expansion of the ILC2 population and accordingly, this inflammation was exacerbated in EP4 deficient mice (148). The production of PGE2 in the lung by alveolar fibroblasts and alveolar macrophages has been widely reported (151, 152), therefore it is not surprising that ILC2s in this tissue express receptors for that mediator. Even though there have not been additional reports of PGE2 regulating ILC2s in other tissues, we cannot rule out that it might impact their function in other compartments.

### Prostaglandin I2 Receptor

The prostaglandin I2 receptor also called prostacyclin receptor or IP is a Gs protein-coupled receptor that increases cAMP levels (150) and can reduce allergic inflammation in different animal models (153, 154).

Expression of IP on ILC2s has been reported in the lung only (**Figure 1**). Stimulation of the IP pathway was shown to be important for the regulation of IL-5 and IL-13 secretion in lung ILC2s *in vivo* in an IP-deficient mouse model of allergic inflammation (155, 156). However, analogs of the IP ligand, PGI2, can regulate ILC2s differentiated from bone marrow by a mechanism involving inhibition of IL-5 and IL-13 production, in response to IL-33, in an IP-dependent manner (155).

### Cysteinyl Leukotriene Receptors

Leukotrienes (LTs) are mediators derived from arachidonic acid involved in self-defense systems, but overproduction causes a variety of inflammatory diseases (157). Leukotrienes (LT) C4, D4 and E4 function as ligands for cysLTRs. So far, five CysLT receptors have been identified: CysLTR1, CysLTR2, P2Y12, GPR99, and GPR17; but only CysLTR1 and CysLTR2 have been reported on ILC2s.

LTD4 has higher affinity than LTC4 to cysLTR1; cysLTR2 is similarly related to these two leukotrienes, with E4 having the lowest affinity for this receptor. CysLTR1 and CysLTR2 are coupled to Gq and Gi proteins, so they exert their action by activating PKC and producing calcium mobilization, or decreasing cAMP levels, respectively (157).

CysLTR1 is expressed in lung and bone marrow ILC2s, where LTD4 induces IL-5 and IL-13 expression similarly to IL-33, in a cysLT1R-dependent manner (158, 159). Interestingly, LTD4 induces the expression of IL-4 in lung ILC2s *in vitro* (158), while *in vivo*, LTD4 administration increases the production of IL-5, IL-13 and the proliferation of ILC2s in a model of allergic inflammation (158). Accordingly, studies on type 2 inflammation models using cysLTR1 or LTC4 deficient mice revealed suppressive effects of these responses in ILC2s (159). Also in the lung, ILC2s are activated by leukotriene C4 in synergy with IL-33, resulting in increased proliferation and expression of IL-5 and IL-13 *in vivo*, in a cysLTR1 and NFAT-dependent manner (160). In addition to the production of IL-5 and IL-13, the activation of ILC2s with IL-7 and IL-33 plus LTC4 or LTD4, can induce IL-17 expression on these cells (161).

cysLTR1 and cysLTR2 are expressed similarly on ILC2s in lamina propria of the small intestine (162). *In vitro* activation of these intestinal ILC2s with LTC4 and LTD4 increases their expression of IL-13 (162). The action of these leukotrienes *in vivo* is important for the activation of ILC2s and the control of helminth infections, but irrelevant in controlling infections by protists (162).

The expression of LTC4 and LTD4 in the lung and small intestine is carried out by epithelial cells (**Figures 1**, **2**). In the intestine, it is specifically produced by Tuft cells (163, 164), whose direct production of LTC4 is important in regulating the function of ILC2s (162). However, leukotriene expression has also been reported in the hematopoietic compartment, associating neutrophils, eosinophils, basophils, mast cells, and macrophages as potential sources (165).

## Neuropeptide Receptors

It is known that the immune system is in constant communication with the nervous system, and both are capable of perceiving and responding to external stimuli. This communication relies on cell-cell interactions, soluble mediators, and occurs in different tissues (166, 167). Recent studies have shown that ILC2s can express receptors for several neuropeptides that can positively or negatively regulate their different functions (167).

### Neuromedin U Receptor 1

Neuromedin U (NMU) is a neuropeptide associated with cholinergic neurons and expressed in different tissues. This neuropeptide exerts its action through NMUR1 and NMUR2 receptors (168), which belong to the 7 transmembrane family and are coupled to Gq proteins, inducing mobilization of intracellular calcium upon their activation (168). ILC2s of small intestine and lung express high levels of the NMUR1 gene (**Figures 1**, **2**) but do not express NMUR2 (114, 169), supporting other reports of NMUR2 expression being restricted to the central nervous system (168).

In the lung, NMU can activate ILC2s by inducing the expression of IL-5 and IL-13 alone or in combination with IL-25 (169, 170). In addition, NMU activation induces proliferation of ILC2s and their expression of amphiregulin and CSF-1; all these responses are dependent on NFAT activation (169). Expression of NMU in the lung is increased in response to helminth infection and ablation of this pathway leads to an inefficient control of the infection (169). NMU can increase the ILC2 response by inducing type 2 inflammation following allergen challenges (170). Interestingly, NMU increases the expression of the neuropeptide calcitonin gene related peptide alpha (α-CGRP) and its receptor, however this same treatment decreases the expression of NMUR1 (114).

Gut ILC2s also express the NMUR1 receptor. Its activation in this tissue induces similar effects as those in the lung in terms of the induced expression of IL-5, IL-13, amphiregulin, CSF-1 and IL-9, leading to proliferation and promotion of a defense response against helminth infections (169, 171).

These pathways are important to ILC2 biology since they allow these cells to communicate with the nervous system. In the small intestine, enteric cholinergic neurons are thought to be the source of NMU, while in the lung it is assumed that local cholinergic neurons could be working as sources of this molecule (169).

### Calcitonin Gene Related Peptide Alpha Receptor

Calcitonin gene related peptide alpha is a highly potent vasoactive peptide characterized by having protective effects at the cardiovascular level. Its receptor is composed of 2 subunits, the calcitonin like receptor (CLR) and the transmembrane protein RAMP1 (172). Downstream, this receptor is coupled to Gs and Gq proteins depending on the cell type in which they are expressed (172). Like NMUR1, the expression of the α-CGRP receptor has been identified in ILC2s from lung and gut (**Figures 1**, **2**).

In the lung, ILC2s express both subunits of the α-CGRP receptor with regulatory effects on the function of these cells (173). In culture, α-CGRP increases IL-5 production together with IL-7, IL-25 and IL-33 but only at early time point stimulations. At later times cell proliferation and the levels of IL-5, IL-13 and amphiregulin decrease (114, 173, 174). *In vivo*, intranasal administration of α-CGRP counteracts the effects of IL-33 on ILC2 activation, which is consistent with the exacerbated inflammatory response of ILC2s in RAMP1 deficient mice (114, 173).

In the small intestine, ILC2s express the components of the α-CGRP receptor at steady state; however, this expression decreases under conditions of type 2 inflammation. *In vitro*, α-CGRP reduces the proliferation of ILC2s as well as the production of IL-13 while increasing IL-5 levels at early time points (175). *In vivo*, α-CGRP antagonizes IL-25-dependent activation of ILC2s (175).

Different cells have been identified to express α-CGRP in the lungs, among them are pulmonary neuroendocrine cells (PNEC) (174), sensory neurons, endothelial cells, and ILC2s themselves (114, 173). The source of α-CGRP in homeostasis *in vivo* was described in the gut as enteric neurons that express choline acetyltransferase (175). All these sources have been characterized by being located spatially close to ILC2s in their respective tissues, further supporting the potential relevance of this neuropeptide for ILC2 regulation and function.

### Vasoactive Intestinal Peptide Receptors 1 and 2

The vasoactive intestinal peptide (VIP) was initially characterized as a potent vasodilator widely distributed in the central and peripheral nervous system, as well as in the digestive, respiratory and cardiovascular systems. Both VIP receptors, VPAC1 and VPAC2, signal through adenylate cyclase and activate the PKA pathway (176).

VPAC1 and VPAC2 are expressed in intestinal ILC2s (**Figure 2**) and their activation induces an increase in the expression of IL-5, which regulates the recruitment of eosinophils in basal conditions (177).

Both receptors are also expressed in lung ILC2s (**Figure 1**) and similarly to what has been described in the intestine, VIP-mediated activation increases IL-5 expression (177, 178). IL-5 then stimulates nociceptor neurons in the tissue to produce VIP, thus forming a positive regulatory loop with ILC2s in a context of allergic inflammation (178).

Like other neuropeptides, VIP is mainly expressed by neurons that innervate these tissues; therefore, the expression of all these receptors and their ligands licenses a complex system for the regulation of ILC2 function controlled by the nervous system.

### Beta 2 Adrenergic Receptor

Upon ligand binding, adrenergic receptors can activate G proteins. In the case of β2AR, the binding of adrenaline and norepinephrine induces the activation of Gs proteins that in turn increase cAMP levels and activate the PKA pathway. It should be noticed that the same receptor has also been reported to activate Gi proteins (179).

The expression of beta 2 adrenergic receptor (β2AR) has been described in ILC2s of the small intestine, colon and lung (**Figures 1**, **2**) (180). In the small intestine and lung, binding to its ligand results in a negative regulation of ILC2 activation. β2AR-deficient mice showed increased ILC2 proliferation in a helminth infection model while β2AR agonist treatment of small intestine ILC2s decrease cytokine expression (180). Similar effects were observed in the lung using sterile inflammation models such as intranasal administration of IL-33 or challenge with allergens (180).

Expression of the β2AR ligands have been reported in both lung and small intestine. Sympathetic adrenergic neurons that are in regions anatomically relevant within these tissues may be inhibiting the action of ILC2s by secreting epinephrine and norepinephrine (180).

### Acetylcholine Receptors

Acetylcholine (ACh) exerts its effects activating two groups of receptors: nicotinic and muscarinic. The nicotinic receptors are pentameric and function as sodium ion channels that activate neurons (181), while muscarinic are G protein-coupled receptors classified in 5 types (M1-M5): M1, M3 and M5 are activator receptors associated with G_q/11_ proteins. while M2 and M4 are inhibitory receptors associated with G_i/o_ proteins (182).

The α 7 nicotinic acetylcholine receptor (α7nAChR) is an excitatory synaptic receptor that serves as a therapeutic target in different neurological and inflammatory disorders (183). Expression of α7nAChR has been reported in lung ILC2s after intranasal administration of IL-25 and IL-33. Acetylcholine receptor agonists can attenuate the type 2 immune response in pulmonary allergic inflammation models (184). Treatment with agonists of this receptor *in vitro* and *in vivo*, decreases the expression of IL-5 and IL-13 induced by IL-33. Cholinergic activation has also been reported to inhibit the function of ILC2s in airway hyperreactivity models and in challenges with *Alternaria* extract, by inhibiting GATA-3 expression and NFKb activation (183). Contrastingly, in *N. brasiliensis* infection, the administration of ACh agonists, increases the number of IL-5 and IL-13 producing ILC2s in the lung (185).

Small intestine ILC2s express transcripts of the Chrm4 and Chrm5 subunits, associated with muscarinic receptors, as well as the Chrna2, Chrna5, Chrna9, Chrna10, Chrnb1 and Chrnb2 subunits, associated with nicotinic receptors (185). *In vitro* stimulation of these ILC2s increases the expression of IL-5 and IL-13 and experiments with specific inhibitors of both types of receptors, showed that both contribute to the activation of ILC2s mediated by ACh (185).

In addition to cholinergic neurons, it has been proposed that ILC2s produce ACh since the enzyme choline acetyltransferase (ChAT) is expressed in ILC2 of the lung and small intestine during *N. brasiliensis* infection (185, 186). This implies, in addition to an interaction with neurons, an autocrine loop of regulation of the ILC2s through ACh.

#### 5-Hydroxytryptamine 1B Receptor

5-Hydroxytryptamine (serotonin) is perhaps best known as a neurotransmitter that modulates neural activity and a wide range of neuropsychological processes. The 5-HT1B receptor is a receptor with 7 transmembrane domains that is coupled to Gi proteins, therefore its activation results in the inhibition of adenylate cyclase (187).

A recent study showed that ILC2s of mesenteric lymph nodes express the 5-TH1B receptor and tryptophan hydroxylase 1 (Tph1), the limiting enzyme in serotonin biosynthesis (62). Induction of Tph1 in helminth infection is dependent on IL-33 and is important for the generation of iILC2s and the immune response against the parasites (62).

Although it has been reported that the main source of serotonin in the gastrointestinal tract are enterochromaffin cells (188), the finding that ILC2 express Tph1 postulates them as additional sources of this neurotransmitter. In addition, expression of the 5-HT1B receptor in these cells suggests that serotonin can play a role as an autocrine regulator of ILC2s like other mediators such as acetylcholine or IL-9.

## Sex Hormones: Androgen Receptor

Cumulative evidence shows a higher prevalence of asthma in women compared to men (189). This assertion was the starting point to investigate the mechanisms behind this gender-bias, including the study and characterization of sex hormones and how they could be acting on the immune cells associated with this type of inflammation. In general, it has been observed that females have a higher number of ILC2s compared to males (189–191), which could contribute to their predisposition to asthma.

The expression of AR in ILC2 precursors was identified while studying the function of ILC2s in the bone marrow. Subsequent culture of these precursors in the presence of dihydrotestosterone (DHT) showed that this hormone can inhibit ILC2 expansion *in vitro* while an AR antagonist is capable of reversing this effect (190).

In the lung of gonadectomized mice, DHT tests performed *in vivo* and *in vitro* showed that androgens are capable of inhibiting IL-2 dependent proliferation of ILC2s as well as expression of GATA-3, RORα, CD25, KLRG1, IL-5 and IL -13 (189–191). Another study showed that activation of the AR increases Klrg1 expression, which has an inhibitory function when interacting with E-cadherin (113). In androgen receptor deficient mice, ILC2s were regulated in the lung and bone marrow (189–191).

## Nutrients

Given that ILCs participate in the maintenance of tissue homeostasis, it is not surprising that these cells play a role in the regulation of metabolic processes and glucose tolerance (192). How the function of ILCs is regulated by the availability of nutrients and factors derived from diet has been a topic of study in recent years (192).

### Vitamin A

Vitamin A deficiency (VAD) in mice leads to increased expression of IL-5, IL-13 and IL-4 by ILC2s in the small intestine upon helminth infection. In addition, VAD increases proliferation and differentiation of ILC2s and their precursors by augmenting IL-7 receptor expression on these cells, enhancing their responsiveness to IL-7 (193). This deficiency can also lead to a decrease in ILC3 numbers and IL-17 and IL-22 expression (193). These effects occur *via* retinoic acid (RA), a vitamin A metabolite that negatively regulates ILC2s while favoring the function and expansion of ILC3s as a result of the expression of the alpha receptor of RA (RARα) on both cell types (193). This has also been observed in lung, where vitamin A deficiency increases the type 2 immune response, ILC2 activation and the severity of a lung cancer model (194).

The effect of retinoic acid on peripheral blood ILC2s in humans is opposite to that observed in murine tissues. *In vitro* stimulation of ILC2s with retinoic acid synergizes with other cytokines such as IL-7 and IL-33 to enhance IL-5 expression, while in combination with IL-2, IL-7, TSLP, IL-25 and IL-33 it increases the expression of IL-13 (195). Finally, it was also reported that treatment with retinoic acid and IL-2 induces the expression of the gut-homing integrin α4β7 (195). Hence, the effects of retinoic acid differ in humans and mice. Whereas in humans the retinoic acid activates ILC2s in mice the evidence suggests a direct effect through the receptor of this metabolite that regulates the function of ILC2s.

### Fatty Acids

Several reports indicate that ILC2s have a higher fatty acid internalization compared to other cell populations such as regulatory T cells, ILC1s and ILC3s; this specific feature persists in different tissues including the small intestine, lung, skin and mesenteric adipose tissue (196). In the small intestine, the accumulation of ILC2s and production of IL-13 upon helminth infection or malnutrition conditions are dependent on fatty acids oxidation (FAO), as a process required to obtaining energy (196). Lung ILC2s express the free fatty acid receptors FFAR1 and FFAR4 (**Figure 1**), and *in vitro* addition of linoleic acid to IL-33 activated ILC2s, increases the expression of IL-5 (197).

Butyrate, a short-chain fatty acid (SCFA) produced by the fermentation of dietary fibers by the commensal microbiota, has been reported to work as a regulator of ILC2 function *in vivo* (198, 199). ILC2s can also be regulated by butyrate *in vitro*, having inhibitory effects on IL-33-induced expression of GATA-3, IL-5, IL-13 and GM-CSF, as well as on ILC2 proliferation (198, 199). Interestingly, this fatty acid is capable of inducing IL-17 production in ILC2s *in vitro*, decreasing in turn the expression of IL-5 and IL-13. Of note, ILC2s can express low levels of IL-17 in the absence of butyrate, so this SCFA only increases this capacity (198). In an airway hyperreactivity model, the transfer of ILC2s treated with butyrate fails to recruit eosinophils, recruiting neutrophils to the lungs instead (198). Finally, oral butyrate administration decreases ILC2 accumulation in models of lung inflammation (198, 199).

Together, these reports indicate that direct contact with metabolites derived from the diet can modulate the function of ILC2s, not only in the gastrointestinal tract but also in other tissues and this regulation is necessary for the control of the immune response promoted by these cells in different inflammatory contexts.

## ILC2s in Tissues

While the study of ILC subsets is clearly an area of huge interest in the scientific community, most of cumulative data so far on ILC2s are limited to lung, skin and intestine, which are the main sites of inflammation in the canonical models for the study of these cells.

### Lung ILC2s

Many cytokine receptors are expressed in the lung ILC2s (**Figure 1**). This includes receptors for IL-33, IL-25, TSLP, IL-2, IL-4, IL-7, IL-9 and SCF, all of which activate or enhance the activation of ILC2s. Cell-cell interaction molecules also regulate ILC2s in the lung; ICOS provides activation signals while Klrg1 could act as a negative regulator as recently suggested (113). Lung ILC2s also express lipid mediator receptors that transduce signals for prostaglandins and leukotrienes. The activation of CRTH2 and cysLTR1 favor the function of lung ILC2s while signaling through PGE2 and PGI2 receptors negatively regulate them. The neuropeptides NMU and VIP activate ILC2s through their cognate receptors, while some catecholamines have the opposite effect, and CGRP, acetylcholine and fatty acid derivatives provide both positive and negative regulation to ILC2 function. Finally, among factors derived from the diet, retinoic acid functions as a negative regulator of these cells. All these signals allow ILC2s to interact both with other cells of the immune, nervous and epithelial system, as well as with other ILC2s through autocrine regulation by IL-9 or ACh in the lung (**Figure 1**).

### Gut ILC2s

ILC2 communication with the epithelium, and the nervous and immune systems also occurs in the gut (**Figure 2**). In this tissue, the cytokines IL-33, IL-25, IL-7, and IL-9, leukotrienes C4 and D4, and neuropeptides NMU, VIP and ACh contribute to the activation of ILC2s through their cognate receptors, as does the signaling mediated by ICOS and its ligand ICOS-L. Metabolism of fatty acids also favors gut ILC2s, however, to our knowledge there are no reports indicating that it occurs through a receptor. Similarly to the lung, a dual function of CGRP has been reported in the gut, while the B2AR receptor and retinoic acid are related to a negative regulation of ILC2s in this tissue. Klrg1 is also expressed on ILC2s in the gut, although its specific function in this tissue has yet to be characterized (**Figure 2**). Although the interaction of gut ILC2s with different cell groups within the immune, epithelial, and nervous systems are important, a particular interaction that should be noted is that of small intestine ILC2s with Tuft cells. IL-17RB expression is critical in small intestine ILC2s and Tuft cells are the main source of IL-25 and lipid mediators that can activate ILC2s including leukotrienes and prostaglandins.

### Skin ILC2s

Unlike the two previous tissues, Klrg1 in skin has been characterized as a negative regulator of ILC2 function since the interaction of ILC2s with E-cadherin results in a down regulation of the expression of GATA-3 and type 2 cytokines. Conversely, IL-33, IL-25, TSLP, IL-2, IL-4, IL-7, IL-18, and PGD2 provide activating signals to ILC2s through their receptors. The expression of these receptors allows ILC2s to communicate with different cells of the skin microenvironment like fibroblasts and keratinocytes, which are sources of alarmins in the skin, as well as with cytokine-producing granulocytes such as mast cells (**Figure 3**).

## Concluding Remarks

This review summarizes the most current reports associated with the function of a large number of markers, ligands and signals that could help in our understanding of the intricate way ILC2s are regulated and perform their functions *in vivo*, both in homeostasis and in inflammatory models. We are aware that this is a growing field, and new signals able to regulate these cells are being constantly identified. The main focus was on widely described receptors that activate ILC2s as well as recent discoveries of other signals capable of regulating these cells in contexts of inflammation and infection while excluding markers that although are of great importance, have already been covered in other reviews (109, 140, 200–202).

In this review we focus on markers described in both mice and humans, however there is more information associated with ILC2s from tissues in mice given the existing limitations for its study in humans.

In addition to describing the function of different receptors expressed on ILC2s, this review seeks to be a tool to facilitate the use of markers for ILC2 identification in different study models in a tissue-dependent manner. Therefore, despite not knowing the function of some of the molecules mentioned, in **Table 1** we summarize their expression reported at the protein level in different models. In addition to the lung, gut and skin ILC2 markers, we include markers for their identification in adipose tissue and bone marrow. ILC2s have been reported to be present at different anatomical locations within adipose tissue and are important for the maintenance of its homeostasis. Multiple studies of ILC2 precursors have been carried out in the bone marrow and the ILC2s from this lymphoid organ can contribute to the different responses of these cells.

**Table 1:** Expression of markers associated with the identification and regulation of ILC2s in different tissues and study models.

Tissue	Marker	Models	References
**Lung**	ST2	Basal conditions (mouse and human)	(9, 17, 33, 39, 54, 79, 85, 86, 112, 117, 119, 122, 123, 134, 144, 145, 177, 183, 186, 191, 199)
		Allergic inflammation (papain administration)	(53, 105)
		Helminth infection (*N. brasiliensis*)	(54, 92, 123, 185, 186)
		Cecal ligation and puncture sepsis model	(56)
		Intraperitoneal administration of IL-33	(17, 145)
		Intraperitoneal administration of IL-25	(17, 173)
		Intranasal administration of TSLP	(79)
		Intranasal administration of IL-33	(112, 145, 148, 183)
		*A. fumigatus* infection	(85)
		Respiratory syncytial virus infection	(134)
		*Alternaria alternata* challenge	(148, 199)
	IL-17RB	Basal conditions (mouse and human)	(58, 94, 186)
		Intraperitoneal administration of IL-25	(17)
		Helminth infection (*N. brasiliensis*)	(186)
	TSLPR	Basal conditions	(78–80)
		Respiratory syncytial virus infection	(78)
		Intranasal administration of IL-33	(79)
	IL-2Rα	Basal conditions (mouse and human)	(33, 58, 84, 85, 93, 94, 107, 112, 119, 122, 177, 183, 189–191)
		Allergic inflammation (HDM* administration)	(84)
		Helminth infection (*N. brasiliensis*)	(27, 92)
		Intranasal administration of IL-33	(84, 148, 183)
		*A. fumigatus* infection	(85)
		Allergic inflammation (papain administration)	(93, 105)
		*Alternaria alternata* challenge	(148, 197)
	IL-4Rα	Basal conditions (mouse and human)	(93, 94)
		Allergic inflammation (papain administration)	(93)
		Helminth infection (*N. brasiliensis*)	(92)
	IL-7Rα	Basal conditions (mouse and human)	(19, 33, 58, 61, 84, 86, 93, 118, 119, 122, 144, 145, 177, 185, 189, 199)
		Helminth infection (*N. brasiliensis*)	(27, 92, 185)
		Intraperitoneal administration of IL-25	(17)
		Allergic inflammation (HDM* administration)	(84)
		Allergic inflammation (papain administration)	(93)
		Intraperitoneal administration of IL-33	(145, 185)
		Intranasal administration of IL-33	(145, 180, 183)
		*Alternaria alternata* challenge	(199)
	IL-9Rα	Basal conditions (human)	(94)
	IL-18R1	Basal conditions	(39)
	Klrg1	Basal conditions (mouse and human)	(19, 84, 86, 112, 114, 118, 156, 170, 177, 190, 191)
		Intraperitoneal administration of IL-25	(17, 173)
		Intranasal administration of IL-33	(84, 112, 156, 170)
		Allergic inflammation (HDM* administration)	(84)
		Helminth infection (*N. brasiliensis*)	(17, 92, 123)
		Intranasal administration of IL-25	(170)
	ICOS	Basal conditions (mouse and human)	(33, 94, 116, 119, 122, 123, 177, 199)
		Intranasal administration of IL-33	(84)
		Allergic inflammation (HDM* administration)	(84)
		Helminth infection (*N. brasiliensis*)	(92, 123)
		Allergic inflammation (papain administration)	(116)
		*Alternaria alternata* challenge	(199)
	ICOS-L	Basal conditions	(20)
	MHC-II	Basal conditions	(122)
		Helminth infection (*N. brasiliensis*)	(123)
		Allergic inflammation (papain administration)	(105)
	KIT	Basal conditions	(33, 84, 133, 134)
		Influenza A virus infection	(133)
		Respiratory syncytial virus	(134)
		Intraperitoneal administration of IL-25	(17)
		Allergic inflammation (HDM* administration)	(84)
	CRTH2	Basal conditions (human)	(94)
	IP	Basal conditions	(155)
	CysLTR1	Basal conditions	(158)
		*Alternaria alternata* challenge	(158)
	NMUR1	Basal conditions	(170)
		Intranasal administration of IL-25	(170)
	α7nAChR	Intranasal administration of IL-33	(183)
		Intranasal administration of IL-25	(183)
	Thy1	Basal conditions (mouse and human)	(33, 39, 56, 85, 86, 93, 117, 143–145, 158, 177, 183, 189–191, 193, 197, 199)
		Cecal ligation and puncture sepsis model	(56)
		Intraperitoneal administration of IL-25	(17)
		Helminth infection (*N. brasiliensis*)	(17, 27)
		*A. fumigatus* infection	(85)
		Allergic inflammation (papain administration)	(93, 105)
		Intraperitoneal administration of IL-33	(145, 190)
		Intranasal administration of IL-33	(145, 180)
		*Alternaria alternata* challenge	(158, 197, 199)
	Sca-1	Basal conditions (mouse and human)	(33, 86, 107, 133, 156)
		Intraperitoneal administration of IL-25	(17)
		Allergic inflammation (papain administration)	(105)
		Intranasal administration of IL-33	(105, 156)
		Intranasal administration of IL-25	(105)
		Influenza A virus infection	(133)
		*Alternaria alternata* challenge	(197)
**Gut**	ST2	Basal conditions	(60–62, 72, 107, 118)
		Intraperitoneal administration of IL-33	(63)
		Helminth infection (*T. spiralis*)	(74)
	IL-17RB	Basal conditions	(39, 61, 72)
		Tritrichomonas colonization	(72)
		Helminth infection (*T. spiralis*)	(74)
	IL-2Rα	Basal conditions	(63, 107, 112, 118, 119)
		Intraperitoneal administration of IL-33	(63)
	IL-7Rα	Basal conditions	(61, 107, 112, 118, 119, 171, 177)
		Intraperitoneal administration of IL-33	(63)
		Helminth infection (*T. spiralis*)	(74)
	Klrg1	Basal conditions	(60–62, 72, 107, 114, 118, 119, 162, 171, 177, 180)
		Tritrichomonas colonization	(72)
		Helminth infection (*T. spiralis*)	(74)
		Helminth infection (*N. brasiliensis*)	(171)
	ICOS	Basal conditions	(118, 119, 177)
	MHC-II	Basal conditions	(74)
		Helminth infection (*T. spiralis*)	(74)
		Intraperitoneal administration of IL-33	(63)
	KIT	Basal conditions	(107)
		Intraperitoneal administration of IL-33	(63)
	CRTH2	Basal conditions	(143)
	NMUR1	Basal conditions	(171)
		Helminth infection (*N. brasiliensis*)	(171)
	Thy1	Basal conditions	(39, 72, 107, 193)
		Intraperitoneal administration of IL-33	(63)
	Sca-1	Basal conditions	(107)
**Skin**	ST2	Basal conditions	(15, 64, 96)
		Wounding model	(64)
		Induced expression of IL-33 in skin	(65)
		Atopic dermatitis (MC903 administration)	(15, 95)
		Atopic dermatitis (human)	(15, 16, 95)
	IL-17RB	Basal conditions	(58)
		Atopic dermatitis (human)	(16)
	TSLPR	Atopic dermatitis (human)	(16)
	IL-2Rα	Basal conditions (mouse and human)	(15, 16, 58, 64, 87, 112)
		Wounding model	(64)
		Atopic dermatitis (MC903 administration)	(15, 95)
		Atopic dermatitis (human)	(15, 95)
		Allergic inflammation (DDAC** administration)	(87)
	IL-4Rα	Basal conditions	(95)
	IL-7Ra	Basal conditions (mouse and human)	(15, 16, 58, 81, 87, 96, 112, 119, 135)
		Atopic dermatitis (human)	(16)
		HDM* administration (Human)	(81)
		Allergic inflammation (DDAC** administration)	(87)
	IL-18R1	Basal conditions	(39)
	Klrg1	Basal conditions	(95)
		Atopic dermatitis (human)	(16)
	ICOS	Basal conditions (mouse and human)	(15, 16, 87, 88, 119)
		Allergic inflammation (DDAC** administration)	(87)
	KIT	Basal conditions (mouse and human)	(15, 16, 129, 135)
	CRTH2	Basal conditions (human)	(16, 81, 96, 129, 135)
		HDM* administration (Human)	(81)
		Allergic inflammation (DDAC** administration)	(87)
		Psoriasis (human)	(135)
	Thy1	Basal conditions	(15, 39, 64, 88, 193)
		Wounding model	(64)
	Sca-1	Basal conditions	(15)
		Induced expression of IL-33 in skin	(65)
**Adipose tissue**	ST2	Basal conditions (mouse and human)	(18, 22, 37, 39, 70)
		Intraperitoneal administration of IL-33	(22)
	IL-2Rα	Basal conditions (mouse and human)	(18, 22, 37)
		Intraperitoneal administration of IL-33	(18)
	IL-7Ra	Basal conditions (mouse and human)	(18, 22, 37, 61)
	Klrg1	Basal conditions	(18, 114)
	ICOS-L	Basal conditions	(20)
	KIT	Basal conditions	(18, 37)
	Thy1	Basal conditions	(18, 37, 39, 70)
	Sca-1	Basal conditions	(18, 37)
**Bone marrow**	ST2	Basal conditions	(39, 59, 84, 191)
		Intranasal administration of IL-33	(59, 84)
		Allergic inflammation (HDM* administration)	(84)
	IL-17RB	Basal conditions	(39, 58)
		Allergic inflammation (*A. alternata*)	(58)
	IL-2Rα	Basal conditions	(58, 59, 84, 190)
		Allergic inflammation (*A. alternata*)	(58)
		Intranasal administration of IL-33	(59, 84)
		Allergic inflammation (HDM* administration)	(84)
		Intraperitoneal administration of IL-33	(145)
	IL-7Ra	Basal conditions	(33, 58, 59, 84, 191)
		Allergic inflammation (*A. alternata*)	(58)
		Intranasal administration of IL-33	(59, 84)
		Allergic inflammation (HDM* administration)	(84)
	Klrg1	Basal conditions	(191)
		Intranasal administration of IL-33	(84)
		Intraperitoneal administration of IL-33	(145)
	ICOS	Basal conditions	(84)
		Intranasal administration of IL-33	(84)
		Allergic inflammation (HDM* administration)	(84)
	KIT	Basal conditions	(33)
	CysLTR1	Basal conditions	(158)
	Thy1	Basal conditions	(39, 158, 191)
		Intraperitoneal administration of IL-33	(145)
	Sca-1	Basal conditions	(33, 190, 191)
		Intraperitoneal administration of IL-33	(145)

^*HDM, House dust mite.^

^**DDAC, Didecyldimethylammonium chloride.^

CD90 and Sca-1 are a couple of markers widely expressed on ILC2s commonly used to identify these cells in most tissues. However, their role in the function of ILC2s is not entirely clear and additional studies are required to fully understand their importance *in vivo* (**Figures 1**–**3** and **Table 1**).

Establishing a panel of specific markers for the identification of ILC2s in different tissues is a complicated task. For example, early reports suggested that alarmin-dependent activation of ILC2s could be tissue-specific. ST2 was found to be expressed on ILC2s of the lung, while in the intestine, the response to IL-25 appeared to be more relevant than IL-33, given the expression of IL-17RB in this tissue. However, current evidence suggests that ILC2s from different tissues have the potential to express the same markers and their profiles are established as a result of modulation by the microenvironment, challenges and interaction with other cell types.

Another complication when selecting a marker to track ILC2s during the course of a study is that their activation modulates the expression of the different markers. In *N. brasiliensis* infection, iILC2s are induced during a specific window of time in the lung and small intestine. These cells are characterized by expressing IL-17RB but not ST2 or CD25. In the lung, iILC2s subsequently give rise to natural ILC2s with increased ST2 expression. For this reason, it is critical to understand how the expression of a marker is expected to change in a specific tissue on different models.

The information presented in this review has been compiled in **Table 1**. At steady state, the markers shared by ILC2s in most tissues include Thy1, IL-7Ra and IL-2Ra. In lung ILC2s, ST2 is one of the more consistently reported markers, while the basal expression of IL-17RB is more commonly used to identify gut ILC2s. Although there are few reports about IL-18R1, it could be an option to identify skin ILC2s, given its differential expression with respect to ILC2s from other tissues. Finally, Klrg1 has been widely reported in the lung and gut ILC2s, not so in the skin.

We would like to emphasize that we do not rule out that some other markers not listed here might be used to identify ILC2s. Future studies could provide additional markers to identify ILC2s in a tissue specific manner, especially those involved in the interaction of these cells with other cell types including epithelial and neural. Another issue to consider is the shared expression of markers such as Thy1 or IL-7Ra with other leukocytes, including other groups of ILCs. Therefore, until specific ILC2 markers are found, the use of ST2, IL-17RB, IL-18R1 or IL-2Ra will require additional lineage cocktails for negative selection.

Even though most of studies have characterized ILC2s in the lung, skin and intestines, new studies indicate that these cells are important in many other organs, performing functions in both homeostasis and inflammation. ILC2s are also located in the stomach, liver and central nervous system. Therefore, it is likely that future studies will describe the presence of ILC2s and novel markers for their identification in different tissues, supporting the heterogeneity of their subsets in a tissue and environmental specific manner.

Interestingly and as mentioned throughout this review, growing evidence suggests that the same pathway can fulfill different functions depending on the context. Therefore, the availability of different signals as well as their sources can substantially vary among distinct tissues. Data discussed in this review, together with cumulative evidence related to particular signals in different tissue-specific microenvironments can help us understand not only how ILC2 are activated but also how they function as part of a more complex system.

Altogether, the information compiled in this review highlights the complexity of ILC2s, which are an extremely important part of the immune response. Not only are ILC2s initiators of inflammatory processes and aid in the maintenance of tissue homeostasis, but they also link the immune system to other systems, allowing a timely and efficient response of the entire organism to different challenges. We also would like to stress that the heterogeneity of ILC2s is highly important and the selection of markers used to identify these cells when working on a specific model should be carefully considered. Acknowledging the differential expression profile of ILC2s specific to each tissue resident population could help our understanding of ILC2s and reconcile apparent controversies between reports of their functions *in vivo*.

## Author Contributions

EO-M conceived the idea, wrote and discussed the review. BR-M revised the manuscript. PL-L conceived the idea, discussed, and revised the manuscript. All authors contributed to the article and approved the submitted version.

## Funding

This work was supported by the following grants to PLL from CONACYT (FORDECYT-PRONACE-303027) and DGAPA (IN209919-PAPIIT). EO-M received a fellowship from CONACYT (481437).

## Conflict of Interest

The authors declare that the research was conducted in the absence of any commercial or financial relationships that could be construed as a potential conflict of interest.

## Publisher’s Note

All claims expressed in this article are solely those of the authors and do not necessarily represent those of their affiliated organizations, or those of the publisher, the editors and the reviewers. Any product that may be evaluated in this article, or claim that may be made by its manufacturer, is not guaranteed or endorsed by the publisher.
